# Interactions between the imperiled West Indian manatee, *Trichechus manatus*, and mosquitoes (Diptera: Culicidae) in Everglades National Park, Florida, USA

**DOI:** 10.1038/s41598-020-69942-8

**Published:** 2020-07-31

**Authors:** Lawrence E. Reeves, Jennifer L. Gillett-Kaufman

**Affiliations:** 1 Florida Medical Entomology Laboratory, Institute of Food and Agricultural Sciences, University of Florida, 200 9th St. SE, Vero Beach, FL 32962 USA; 2 Entomology and Nematology Department, Institute of Food and Agricultural Sciences, University of Florida, 1881 Natural Area Drive, Steinmetz Hall, Gainesville, FL 32611 USA

**Keywords:** Biodiversity, Conservation biology, Ecological epidemiology, Entomology

## Abstract

Arthropod-borne viruses (arboviruses), including those vectored by mosquitoes, have recently been cited as potential emerging health threats to marine mammals. Despite the fully aquatic habits of cetaceans, immunologic exposure to arboviruses including West Nile virus and Eastern equine encephalitis virus has been detected in wild Atlantic bottlenose dolphins, and captive orcas have been killed by West Nile virus and St. Louis encephalitis virus. Currently, there is no evidence of direct interactions between mosquitoes and marine mammals in nature, and it remains unknown how wild cetaceans are exposed to mosquito-vectored pathogens. Here, we report the first evidence of direct interactions between an aquatic mammal, the West Indian manatee, a federally threatened species, and mosquitoes in nature. Observations of manatees in Everglades National Park, Florida, USA, indicate that mosquitoes of three genera, *Aedes*, *Anopheles*, and *Culex* are able to locate and land on surface-active manatees, and at minimum, penetrate and probe manatee epidermis with their mouthparts. Whether mosquitoes can successfully take a blood meal is not known; however, an arbovirus-infected mosquito can inoculate extravascular host tissues with virus-infected saliva during probing. These observations suggest that it is possible for marine mammals to be exposed to mosquito-vectored pathogens through direct interactions with mosquitoes.

## Introduction

The importance of mosquitoes to domesticated animal and human health, as the vectors of disease-causing pathogens, is widely recognized. Less well understood is the importance of mosquitoes in ecosystems and their impact on wildlife health^1^. Female mosquitoes of most species require a blood meal, taken from another animal, in order to obtain proteins necessary to complete egg development^2^. This requirement can facilitate the transmission of diverse pathogens, particularly viruses, protozoa, and helminths, between vertebrate hosts. The host-use patterns of mosquitoes vary by species. Mosquitoes take blood from all major terrestrial vertebrate classes: Amphibia, Aves, Mammalia, and Reptilia, and at least one is an invertebrate specialist of Annelida hosts^3^. Most mosquitoes specialize on particular types (e.g., endo- or ectothermic animals) or classes of host animals, with a few mosquito species that are relative generalists.

Mosquitoes are well known to take blood from host animals that are amphibious or primarily aquatic. Some mosquito species are host specialists of frogs, including ranids that are closely associated with aquatic habitats^4–6^, and fishes, e.g., mudskippers, other gobies, and eels^7,8^. Crocodilians^9,10^, and semi-aquatic snakes and turtles (e.g., *Agkistrodon piscivorus*, *Python bivittatus*, and *Trachemys scripta*) often serve as hosts for opportunistic mosquitoes^11–13^. There is no direct evidence of interactions between mosquitoes and marine mammals in nature, however, mosquito-vectored viruses (e.g., West Nile virus, Eastern equine encephalitis virus, St. Louis encephalitis virus, and Venezuelan equine encephalitis virus) or immunologic evidence of virus infection have been detected in captive orcas^14,15^ and wild Atlantic bottlenose dolphins^16^. In captive orcas maintained in Florida, vulnerability to arboviral infection is thought to be associated with logging behavior. Logging is a behavior that is common among captive orcas, in which an orca spends time floating at the surface. People who work closely with captive orcas have observed mosquitoes landing on exposed dorsal surfaces when the animals express this behavior, and orcas have been killed by the mosquito-vectored viruses West Nile virus and St. Louis encephalitis virus in Texas and Florida, respectively^17^.

Mosquitoes are active throughout the year in southern Florida^18^. In urban and rural areas, *Aedes aegypti* and *Aedes albopictus* are sympatric and serve as vectors of human pathogens (e.g., Dengue virus, Chikungunya virus, and Zika virus)^19^. Although the *Plasmodium* parasites that cause human malaria have been largely eradicated in the state, *Anopheles albimanus*, one of the primary malaria vectors in the Neotropics, occurs in Miami-Dade and Monroe Counties, particularly in coastal areas^20^. The *Culex* (subgenus *Culex*) vectors of West Nile virus and St. Louis encephalitis virus are common throughout much of the state, including the southern counties^20^. Sylvatic areas of southern Florida within the Greater Everglades Ecosystem support the circulation of Everglades virus, a subtype of the Venezuelan equine encephalitis virus, vectored by *Culex* (*Melanoconion*) *cedecei*^21^.

**Figure 1:**
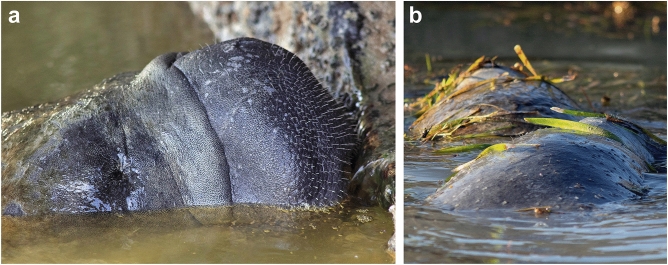
The West Indian manatee, *Trichechus manatus*, frequently spends time at the surface of the water, particularly while drinking freshwater (**a**), or while feeding (**b**). The exposed dorsal surfaces and face are vulnerable to host-seeking mosquitoes, especially when surface activity takes place near-shore, in areas supporting high mosquito abundances such as the Flamingo area of Everglades National Park, Monroe Co., Florida, USA.

In Florida, the West Indian manatee, *Trichechus manatus*, and the Atlantic bottlenose dolphin, *Tursiops truncatus*, occupy areas within or immediately adjacent to salt marshes, mangroves and other habitats that support high mosquito abundance. West Indian manatees are protected in the United States under the Endangered Species Act and Marine Mammal Protection Act; they are listed as a threatened species. In Florida wild and captive manatees are frequently observed at the surface of the water, with their dorsal surface or snout exposed to the air for short periods of time (Fig. 1). Because mosquitoes require a blood meal to reproduce, they have evolved highly effective mechanisms for locating and feeding from hosts. Mosquitoes utilize a variety of cues to locate host animals. Mammalian hosts emit a plume of carbon dioxide and other volatiles that serves as a long-range attractant to many mosquito species^22^. These plumes can be detected and followed by host-seeking mosquitoes at distances of 10–15 m^23^. As mosquitoes approach a host, a combination of acoustic, visual, thermal, olfactory, and humidity cues further guide mosquitoes to their host over shorter ranges^4,24^. Marine mammals that spend time close to shore, particularly near habitats where mosquito populations are dense, would be expected to be attractive to host-seeking mosquitoes. While breathing or at the surface resting, feeding, or drinking they may be vulnerable to mosquito-feeding, and as a result, exposure to mosquito-vectored pathogens, if adequate time is spent at the surface. Here, we report evidence that mosquitoes of three genera (*Aedes*, *Anopheles*, and *Culex*) are, at minimum, capable of locating, landing on, and biting West Indian manatees.

## Results and discussion

**Figure 2:**
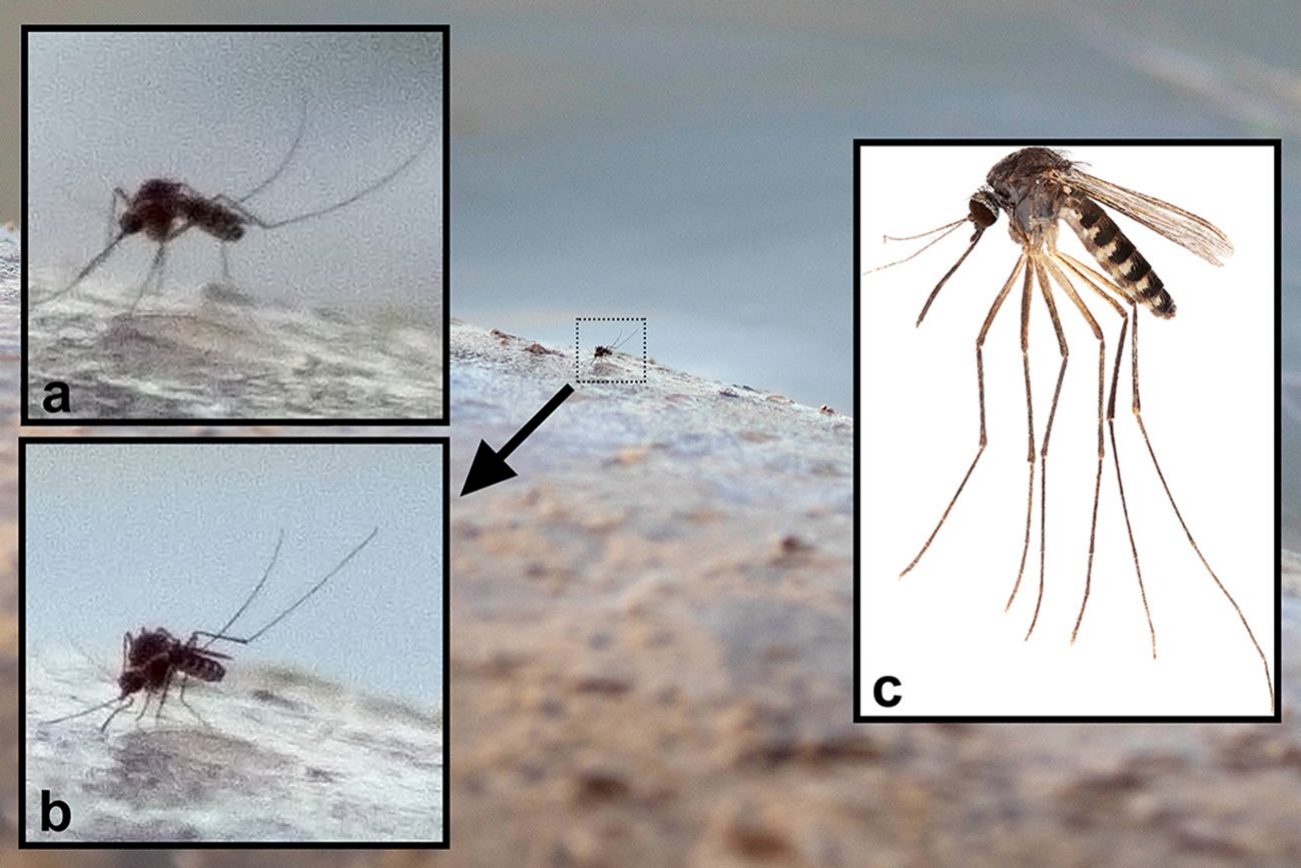
On 16 December 2015 at 0723 h, a mosquito was observed and photographed on the back of a sub-adult West Indian manatee, *Trichechus manatus*, in the Flamingo Marina, Everglades National Park, Monroe Co., Florida, USA. Initially, the mosquito was observed perched upon the back of the manatee (**a**). Subsequent photographs show the reflexed labium of the mosquito (red arrow), presumably with stylets piercing the epidermis of the manatee (**b**). This mosquito can be confidently identified as a species in the *Culex* subgenus *Melanoconion*, possibly *Culex* (*Melanoconion*) *iolambdis* (**c**).

On 16 December 2015, a subadult manatee, < 2 m in length, was observed on the southern (Florida Bay) side of the Flamingo Marina, Everglades National Park, Monroe Co., Florida, resting at the surface with its back exposed above the waterline, approximately 6 m from the concrete edge of the marina. The back of the manatee was inspected for biting flies from a dock through a camera with telephoto lens attached. One mosquito was observed, and a sequence of photographs was taken (Fig. 2). Initially, the mosquito was seen standing on the back of the manatee (Fig. 2a). Subsequent photographs show the labium (the outer sheath of the mosquito proboscis) of the mosquito reflexed, indicating that the stylets (inner mouthparts that pierce skin and locate and feed from blood vessels) were inserted into the skin of the manatee (Fig. 2b). The mosquito remained at the same location on the back of the manatee for 41 s after the first image was taken, until water disturbed by the movement of the manatee flushed the mosquito to flight.

Morphological characters that would enable species-level identification of this mosquito are not visible from the photographs. The visible characters, dark coloration and overall proportions of the mosquito suggest that it is a species of the *Culex* subgenus *Melanoconion*. Six *Melanoconion* species have been documented in Everglades National Park^25,26^, of which two specialize on reptilian hosts, particularly *Anolis* lizards^27^, and one, *Culex mulrennani*, seems to be exceptionally rare^25,26^. Based on the host associations and abundance patterns of these species, the photographed mosquito is likely to be *Culex iolambdis* (Fig. 2c), *Culex erraticus*, or *Culex cedecei*. Both *Culex iolambdis* and *Culex erraticus* are relative host generalists that take blood meals from all terrestrial vertebrate classes^25,28^. *Culex cedecei*, the primary vector of Everglades virus (a strain, Subtype II, of the Venezuelan equine encephalitis virus^29^), is a relative specialist of mammalian hosts, but primarily takes blood meals from rodents in the Everglades^21^.

On 11 July 2017, two adult manatees were observed and photographed at the eastern edge of the southern side of the Flamingo Marina drinking from water flowing into the marina from a concrete pipe. At that time, no mosquitoes were noticed visually or when viewed through the camera. Unlike the observation described above in which there was open water immediately surrounding the manatee, these manatees were located alongside the edge of the marina, and underneath a raised dock where they took turns drinking. One manatee drank as the other jostled for a position at the pipe or floated nearby. The face of the drinking manatee, from above the eyes to the mouth, was above the waterline, but periodically submerged momentarily. Upon later reviewing the images, uploaded to a computer, the presence of at least two individual mosquitoes of different species landing on the snout of the drinking manatee were recognized.

**Figure 3:**
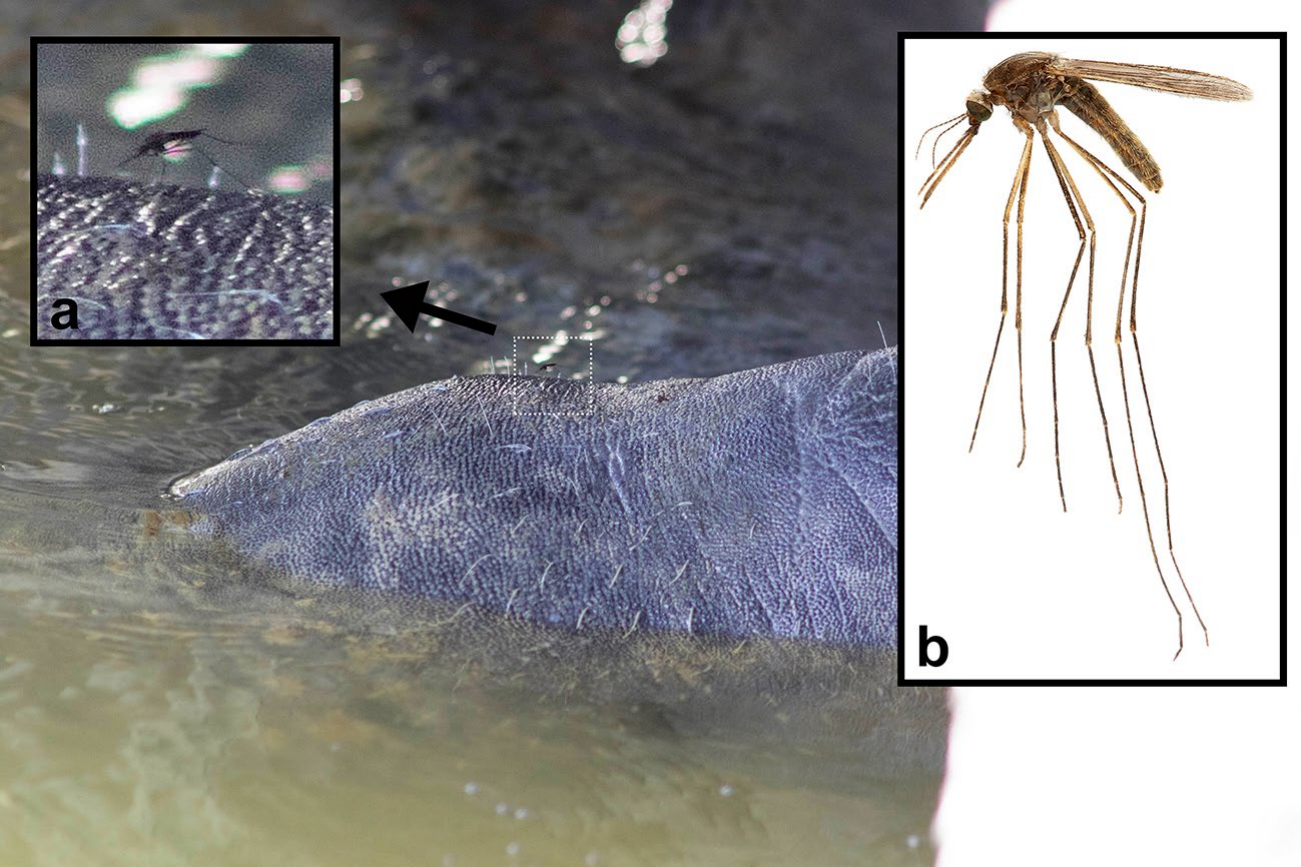
On 11 July 2017 at 1209 h, a manatee was photographed drinking from a pipe on the western edge of the southern side of the Flamingo Marina (Florida Bay), Everglades National Park, Monroe Co., Florida, USA. Upon review of the images, a mosquito was noticed perched above the snout of the manatee, but not apparently feeding (**a**). This mosquito can be confidently identified as a species of *Anopheles*, possibly *Anopheles atropos* (**b**).

The posture and proportions of one of these mosquitoes, observed perching above the eyes of the manatee but not feeding, is typical of the genus *Anopheles*^30^ (Fig. 3a). *Anopheles* mosquitoes are associated with endothermic hosts, and, in general, feed primarily from mammals, and rarely birds. At least six *Anopheles* species are present in extreme southern Florida, including various members of the *Anopheles crucians* and *Anopheles quadrimaculatus* species complexes^20^. In adjacent areas with habitats similar to those around Flamingo, *Anopheles atropos* is the most abundant *Anopheles* species and is among the most abundant mosquito species^31^. Visible characters and coloration are consistent with *Anopheles atropos* (Fig. 3b), but a reliable species-level identification could not be made based on the images.

**Figure 4:**
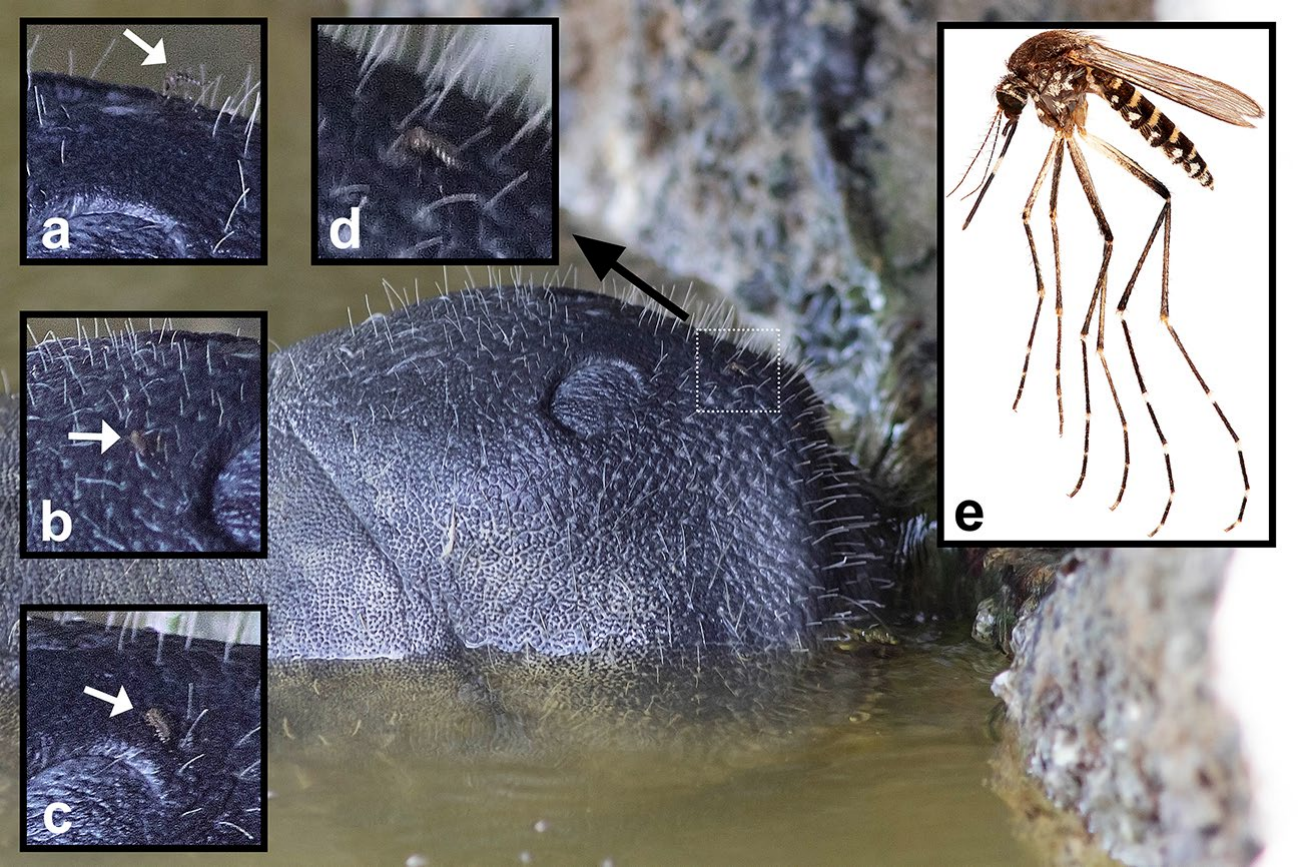
On 11 July 2017 from 1213 to 1216 h, a manatee was photographed drinking from a pipe on the western edge of the southern side of the Flamingo Marina (Florida Bay), Everglades National Park, Monroe Co., Florida, USA. Upon review of the images, a female mosquito, presumably the same individual, was observed at various positions near the nostrils of the manatee (**a**–**d**). This mosquito can be confidently identified as a species of *Aedes*, possibly *Aedes taeniorhynchus* (**e**).

Photographs taken over a 2-min period show another mosquito landing upon the manatee, differing from the first by the presence of white bands on the tarsi of the hind legs (Fig. 4). During this period, the mosquito was photographed at four distinct locations near the nostrils of the manatee (Fig. 4a–d). At each location, the mosquito assumed a feeding posture, with the head and proboscis pointed at the epidermis of the manatee, but the photographs were not of sufficient quality to determine if the labium was reflexed or if the mosquito was successfully feeding. The coloration of the mosquito, particularly the white patterning on the abdomen, brown scutum (dorsal area of the thorax), and white bands on the hindlegs, suggest that this mosquito is either *Aedes taeniorhynchus* (Fig. 4e) or *Aedes sollicitans*. The latter species occupies a more northerly distribution and has not been reported in the literature from Everglades National Park, but is known from Miami-Dade and Monroe Counties^20^. *Aedes taeniorhynchus* is the dominant mosquito species in coastal regions of peninsular Florida and the Everglades, reaching exceptionally high abundance under suitable environmental conditions^32,33^. This mosquito is primarily associated with large- and medium-sized mammalian hosts, and feeds rarely from birds, reptiles, and amphibians in Florida^34^. In the Everglades, *Aedes taeniorhynchus* has been observed feeding in large numbers from American crocodiles^10^. *Aedes sollicitans* has similar host associations in Florida^34^.

The host-use patterns of *Aedes taeniorhynchus* at Flamingo, the same site where the manatee observations were made, were assessed using serology-based blood meal analysis in the 1960s and 1970s^35,36^. These studies found mammals were the dominant hosts (90% of all blood meals attributed to mammalian species), with most blood meals derived from rabbits (73% of identified blood meals) and other medium-sized mammals. Interestingly, 29 blood meals (5%) were determined to be derived from unidentified mammals. Recent work suggests that these host-use patterns have likely shifted, as precipitous declines in populations of medium-sized mammals, including rabbits, the primary hosts at Flamingo, have restructured the community of potential hosts^21^. These declines are attributed to the Burmese python^37,38^, an invasive predator that was likely introduced to the Flamingo area prior to 1985 and has since proliferated throughout the Greater Everglades Ecosystem^39^. Similarly, rising sea levels are expected to dramatically affect the dynamics and availability of larval habitats for salinity tolerant mosquitoes like *Aedes taeniorhynchus*^40^. Because much of the Greater Everglades Ecosystem is at or below sea level, such changes may increase the availability of larval habitats for this, and other saltmarsh mosquitoes in the Everglades.

This is the first evidence of direct interactions between mosquitoes and manatees, or any other fully aquatic mammal, in nature. These observations demonstrate that the aquatic habits of the West Indian manatee do not preclude the species from exposure to mosquitoes, potentially making them vulnerable to mosquito-vectored pathogens. The observation of a female *Culex* mosquito probing a manatee with its labium reflexed (Fig. 2) strongly suggests that mosquito mouthparts are capable of piercing manatee epidermis; however, this does not provide any indication that mosquitoes can successfully locate blood vessels from which to feed. Even without successful blood feeding, an arbovirus-infected mosquito can inoculate extravascular host tissues with virus-infected saliva while probing for blood^41,42^.

To date, exposure to arboviruses has not been documented in manatees, though at least two previous studies have screened manatees for particular arboviruses. While exposure to several mosquito-vectored arboviruses (Eastern equine encephalitis virus, Western equine encephalitis virus, Venezuelan equine encephalitis virus, West Nile virus) has been detected serologically in wild Atlantic bottlenose dolphins from Florida^16^, wild manatees have screened negative for St. Louis encephalitis virus, Western equine encephalitis virus, and West Nile virus in Belize^43^, and for West Nile virus in Florida^44^. Arbovirus infections with fatal and nonfatal outcomes have been documented in captive cetaceans and pinnipeds, which may be at increased risk of exposure to mosquitoes and mosquito-vectored pathogens compared to their wild counterparts^17^. A serological survey for West Nile virus antibodies at a marine park in Texas found individuals of many cetacean and pinniped species to have been exposed to the virus^45^. Fatal infections attributed to St. Louis encephalitis virus^14^ and West Nile virus^15^ in captive orcas have occurred in Florida and Texas, respectively. In captive harbor seals, fatal and nonfatal West Nile virus infections have occurred in Michigan and New Jersey^46^, and a fatal Eastern equine encephalitis virus infection was reported from Massachusetts^47^.

The vector competence (the ability of an arthropod to acquire, maintain and transmit a pathogen) of mosquitoes for a particular pathogen varies from species to species. The mosquitoes observed landing on manatees at the Flamingo Marina can confidently be identified as species of *Aedes*, *Anopheles*, and *Culex*, subgenus *Melanoconion*, all of which include species that are the primary vectors of various pathogens. Based on morphology^20^ and abundance patterns of mosquitoes in Everglades National Park and surrounding areas^26,31^, it is likely that these mosquitoes are *Aedes taeniorhynchus*, *Anopheles atropos* and *Culex iolambdis*. In Florida, mosquitoes of these genera are important in the transmission of pathogens, but none of these possible species are primary vectors for pathogens of known medical or veterinary importance. At the same time, pathogens have been detected in these species: various viruses and parasites have been detected in *Aedes taeniorhynchus* including *Dirofilaria immitis* (dog heartworm), Venezuelan equine encephalitis virus, and West Nile virus in the Florida Keys^48–50^, among others, *Anopheles atropos* is a capable vector for the *Plasmodium* parasites that cause malaria in humans^51^, and Venezuelan equine encephalitis has been detected in *Culex iolambdis*^52,53^.

Manatees and other marine mammals are required to visit the surface to breathe, and to varying extents spend time idling, resting, feeding or drinking at the surface. Satellite- and radio-tracked Atlantic bottlenose dolphins in Florida have been recorded putatively resting at the surface continuously for hours, remaining in one location^54,55^. There is evidence that at least some mosquito species exhibit an avoidance of open water when host-seeking, and as a result, bird species that roost on sandbars, snags in the water or on the water surface are less frequently fed upon and infected by arboviruses^56^. Manatees and other marine mammals that spend time at the surface may be less susceptible to mosquitoes when these behaviors takes place in relatively open water away from shore, but more susceptible if prolonged surface visits take place near the water’s edge, e.g., among dense mangroves, or within narrow canals.

At Flamingo, *Aedes taeniorhynchus* reaches exceptionally high abundances and is a major biting pest of humans during both day and night^32^. In recent CDC light trap (traps that target host-seeking mosquitoes) samples from the Buttonwood Canal, immediately adjacent to the Flamingo Marina, *Aedes taeniorhynchus* outnumbered the next most abundant mosquito by 500 times^26^. Given the dominance of this mosquito at Flamingo, it is noteworthy that only one of three observations of mosquitoes on manatees was likely to be *Aedes taeniorhynchus*. This may indicate that *Aedes taeniorhynchus* avoids host-seeking over open water, which possibly reduces interaction between this mosquito species and surface-resting marine mammals.

Arboviruses have recently been cited as potential emerging pathogens among marine mammals^57–59^. Our observations indicate that direct interactions between mosquitoes of several genera and fully aquatic mammals occur in nature. Photographs associated with these observations demonstrate that mosquitoes are able to land upon, and penetrate manatee epidermis, suggesting that mosquitoes can take blood meals from manatees, and that manatees may be exposed to mosquito-vectored pathogens in nature. Although exposure to mosquito-vectored viruses in aquatic mammals may be the result of undetermined indirect routes of infection, direct mosquito-marine mammal interactions represent the likely route of infection for mosquito-vectored pathogens. Future work should consider that direct interactions between mosquitoes and marine mammals may be common, particularly for animals inhabiting near-shore environments.

## Methods

Observations of manatees were made at the southern side of the Flamingo Marina, Florida Bay, Everglades National Park, Monroe Co., Florida, USA (25° 08′ 31.0ʺ N, 80° 55′ 22.1ʺ W) on 16 December 2015 and 11 July 2017, during research investigating mosquito ecology in the Everglades. Manatees were observed and photographed with a Canon 7D Mk. II digital single-lens reflex (SLR) camera and a 400 mm telephoto lens, without flash. On 16 December 2015 at 0723 h a ~ 2 m subadult manatee was observed for a period of approximately 5 min. Its exposed dorsal surface was inspected through the camera for biting flies, and a sequence of 37 photographs was taken (no flash, ISO 1600, 1/640th s, F5.6). On 11 July 2017 at ~ 1210 h, two adult manatees were observed and photographed (no flash, ISO 1250, 1/250th s, F5.6) for approximately 8 min while drinking from water flowing out of a concrete pipe at the southern (Florida Bay) side of the Flamingo Marina. These individuals were present at the pipe prior to and following the observations.

## Data Availability

No datasets were generated or analyzed during the current study.
